# Heart transplantation surgery in children and young adults with congenital heart disease

**DOI:** 10.1186/s13019-023-02461-5

**Published:** 2023-11-27

**Authors:** Sabrina Martens, Hongtao Tie, Hans Gerd Kehl, Tonny DT Tjan, Hans Heinrich Scheld, Sven Martens, Andreas Hoffmeier

**Affiliations:** 1 Department of Cardiothoracic Surgery, University Hospital Muenster, Albert-Schweitzer-Campus 1, 48149 Muenster, Germany; 2 Department of Pediatric Cardiology, University Hospital Muenster, Albert-Schweitzer-Campus 1, 48149 Muenster, Germany

**Keywords:** Congenital Heart Disease, Heart transplantation

## Abstract

**Background:**

Pediatric cardiac transplantation remains a surgical challenge as a variety of cardiac and vessel malformation are present in patients with congenital heart disease (CHD). Despite limited availability and acceptability of donor hearts, the number of heart transplantations remains on a stable level with improved survival and quality of life.

**Observation:**

As treatment options for CHD continue to improve and the chances of survival increase, more adult CHD patients are listed for transplantation. This review focuses on the clinical challenges and modified techniques of pediatric heart transplantations.

**Conclusion:**

Not only knowledge of the exact anatomy, but above all careful planning, interdisciplinary cooperation and surgical experience are prerequisites for surgical success.

## Introduction

Congenital heart defects (CHD) are the most common malformations in humans − 0.8% of live births are affected in Europe [1]. There are currently around 300,000 CHD patients in Germany, and 95% of them will persist into adulthood [1]. Despite great improvements in surgical techniques and peri-/postoperative care, these patients are still suffering from chronic illness. Heart transplantation can be indicated in both the early or long-term course [2]. Especially patients for whom neither corrective nor palliative surgical procedures are available are transplant candidates, as well as patients with end-stage heart failure and cardiomyopathies.

The first clinical heart transplant was accomplished in an adult by Barnard in Cape Town, South Africa, in 1967 [3]. 3 days later, Kantrowitz attempted the first pediatric heart transplant in the United States. The operation was performed without a heart-lung machine, only in deep hypothermia (17 °C). The 19-day-old child with severe Ebstein malformation died 6 h after the operation in severe metabolic and respiratory acidosis [4]. In Germany, the first heart transplant on an adult with congenital heart disease was carried out by Sebening in Munich in 1968. Despite a technically successful operation, the patient died 27 h after transplant due to an acute occlusion of the right coronary artery [5]. Scheld’s group performed neonatal and pediatric heart transplants in Giessen [6]. The first neonate who had a transplant is currently still alive [7, 8].

Since 1982, more than 14,000 heart transplants have been performed in pediatric patients worldwide [8], which corresponds to around 10% of all heart transplants [9]. About 50% of pediatric patients received heart transplants due to CHD [8], while this proportion is 2.2% in the adult comparison group [10, 11]. In this study, we focus on the complex surgical techniques of heart transplantation in children and young adults and present our own experiences with this special group of transplant candidates.

We investigated not only the perioperative outcome, but also paid special attention to their long-term survival. All CHD patients who underwent transplantation in our institution since 1990 were included. Our local ethics committee approved this study, written informed consent was obtained from all participating patients.

Baseline as well as intra- and postoperative variables are summarized in Table 1.

**Table 1:** Demographic, intra-operative, post-operative, follow-up data

Variables	Median (Range) / n (%)
**Demographic data**	
Age (years)	7.75 (0.11-42)
Male gender	9 (45%)
Weight (kg)	19.1 (3–77)
Length (cm)	11.2 (49–188)
**Main diagnosis**	
Aortic valvular disease	4 (20%)
Ebstein’s anomaly	1 (5%)
VSD, PH	1 (5%)
Single ventricle defects	4 (20%)
TOF	1 (5%)
TGA	4 (20%)
BWG	3 (15%)
DORV	1 (5%)
DCM, mitral regurgitation	1 (5%)
Preoperative VAD-support	6 (30%)
**Intra-operative data**	
Donor heart ischemic time (minutes)	240 (123–359)
Bypass time (minutes)	214 (98–427)
Cross-clamp time (minutes)	80 (41–186)
Surgery time (minutes)	325 (225–659)
Reconstruction procedure	7 (35%)
**Postoperative data**	
Postoperative cardiac re-operations*	9 (45%)
Postoperative neurologic event	5 (25%)
Postoperative sepsis	4 (20%)
Postoperative bleeding	4 (20%)
Postoperative SIRS	2 (10%)
Postoperative renal failure	3 (15%)
Postoperative hepatic dysfunction	3 (15%)
Right ventricular failure	0
Hight-output failure	0
Low-output failure	2 (10%)
30-day mortality	4 (20%)

AI/AS: aortic insufficiency/stenosis; ASD: atrial septal defect; AVC/R: aortic valve commissurotomy/reconstruction; BWG: Bland-White-Garland syndrome; (cc/d)-TGA: (congenitally-corrected/dextro)-transposition of great arteries; CHD: congenital heart disease; DCM: dilatative cardiomyopathy; DORV: double outlet right ventricle; ECLS: extracorporeal life support; EFE: endocardial fibroelastosis; HLHS: hypoplastic left heart syndrome; LVAD: left ventricular assist device; m: months, MCS: mechanical circulatory support; MI: mitral insufficiency; PDA: persistent ductus arteriosus; PH: pulmonary hypertension; PM: pacemaker; PVA: pulmonary valve atresia; SV: single ventricle; TI: tricuspid insufficiency; TOF: Tetralogy of Fallot; VSD: ventricular septal defect; y: years

## Preoperative characteristics of CHD patients before transplant

Depending on the natural courses of different underlying CHD pathologies, most potential transplant candidates are between the ages of 20 and 40, with the most common congenital malformation of TGAs [12].

90% of CHD patients have endured multiple prior cardiac surgeries [13, 14]. These prior procedures and related interventions, including tissue allograft or patch implantation, ventricular assist devices support, and (multiple) blood transfusions, were associated with increased sensitization susceptibility [15, 16]. Besides, due to multiple organ dysfunctions, patients with single ventricle and palliated with Fontan are at high risk for early mortality while on the waiting list [17]. With the development of interventional cardiology and cardiac surgery, more than 90% of CHD patients are alive at 16 years of age, leading to a growing adulthood population of CHD patients [18]. Heart failure in the long-run is the leading death cause in this population. CHD patients who are refractory to medical therapy remain a challenge for clinicians, and cardiac transplantation may be considered as the last treatment option [19].

## Patient selection and preparation

Heart transplantation in CHD patients poses a surgical challenge, especially after multiple previous heart operations and anatomical anomalies of the heart and blood vessels. Meticulous planning, interdisciplinary collaboration, and extensive surgical experience are pre-requisites to establish stable circulatory systems in recipients [20, 21].

Potential recipients must be selected carefully, with the same strict criteria for non-CHD recipients. Particularly in patients with HLHS, staged surgical palliation has achieved outstanding successes in the last 20 years, and thus the number of heart transplants is decreasing more and more due to this indication [22]. The heart transplantation in CHD patients is usually complicated by multiple previous operations, cardiac defects, abnormal situs, and collateral circulation, which should be precisely recorded before the heart transplantation. Schematic illustrations of the initial anatomical situation were helpful to plan the transplantation technique and prepare the operation optimally. Preoperative determination of the pulmonary vascular resistance was also essential, as well as the knowledge about intracardiac shunts and collateral circulation [23].

International guidelines help with patient selection and recommend both non-invasive (regular echocardiography, MR or CT etc.) invasive diagnostics prior to listing for transplantation. Cardiac output can be assessed by spiroergometry (maximal oxygen uptake (peak VO_2_) ≤ 12 ml O_2_/kg bw/min). Cardiac catheterization with calculation of pulmonary arterial resistance can be used for hemodynamic assessment. Fixed pulmonary hypertension (PVR ≥ 8 WU x m^2^ and resistance ratio Rp/Rs > 0.5, transpulmonary gradient > 15 mmHg) need to be considered a contraindication for transplantation [24].

## Circulatory support in patients with CHD

When transplant programs were introduced worldwide, no circulatory assist devices were available for children. Later, mechanical circulatory support (MCS) was limited to Novacor and Medos ventricular assist devices before the Berlin Heart® device was launched. In 2010, our working group reported about a neonate who needed circulatory support with an Excor System (Berlin Heart®) for 452 days before successful transplantation [25]. Increasing life expectancy due to improved ventricular assist devices implies that many CHD patients are supported with a VAD system before heart transplantation.

Both adult and pediatric CHD patients are at a high risk of waiting list mortality or delisting because of clinical deterioration [12, 26]. Early transplantation and MCS should be encouraged; however, median waiting time is around 8 months [12]. Though VAD has been widely used for end-stage heart failure and bridge-to-transplantation or destination therapy, VAD application in CHD patients is not extensively used [27]. Complex cardiac anatomies, abnormal physiology, multiple prior cardiac surgeries, and comorbidities, hinder VAD application. Since the device is used for morphological left ventricles, its application in CHD patients with single ventricles, morphological systemic right ventricle, or failing Fontan with increased pulmonary is limited [28]. Despite these challenges, VADs in pediatric and adult CHD populations are increasing due to the shortage of donor’s hearts and an increasing number of patients in heart failure [29, 30].

According to the pediatric report of the European Registry for Patients with Mechanical Circulatory Support, 461 patiens required VAD support (446 LVAD/BiVAD; 10 RVAD; 2 TAH; 3 unknown) between 2001 and 2020. MCS provided long-term support and was used as bridge-to-transplant (n = 291) or possible bridge-to-transplant (n = 117). Mainly the Berlin Heart Excor system was implanted (n = 246; 53.4%) [31].

Previous studies found that VAD therapy can prolong survival in high-risk CHD patients who would either die or be delisted due to clinical deterioration [32]. For post-transplant outcomes, VAD support before transplantation is reported to be associated with longer post-transplant hospital and ICU length of stay and increased probability of transfusion in adult CHD patients. Nonetheless, the 30-day mortality (10.8% vs. 13.5%, p = 0.62) and overall survival (log-rank p = = 0.57) was similar between adult CHD patients with and without VAD support before transplantation [33]. Another study also showed comparable post-transplant outcomes in pediatric and adult CHD patients with and without VAD support [25]. CHD patients who receive VAD support have a poor medical status, which implies that VAD support can mitigate certain risk factors before transplantation.

**Fig. 1:**
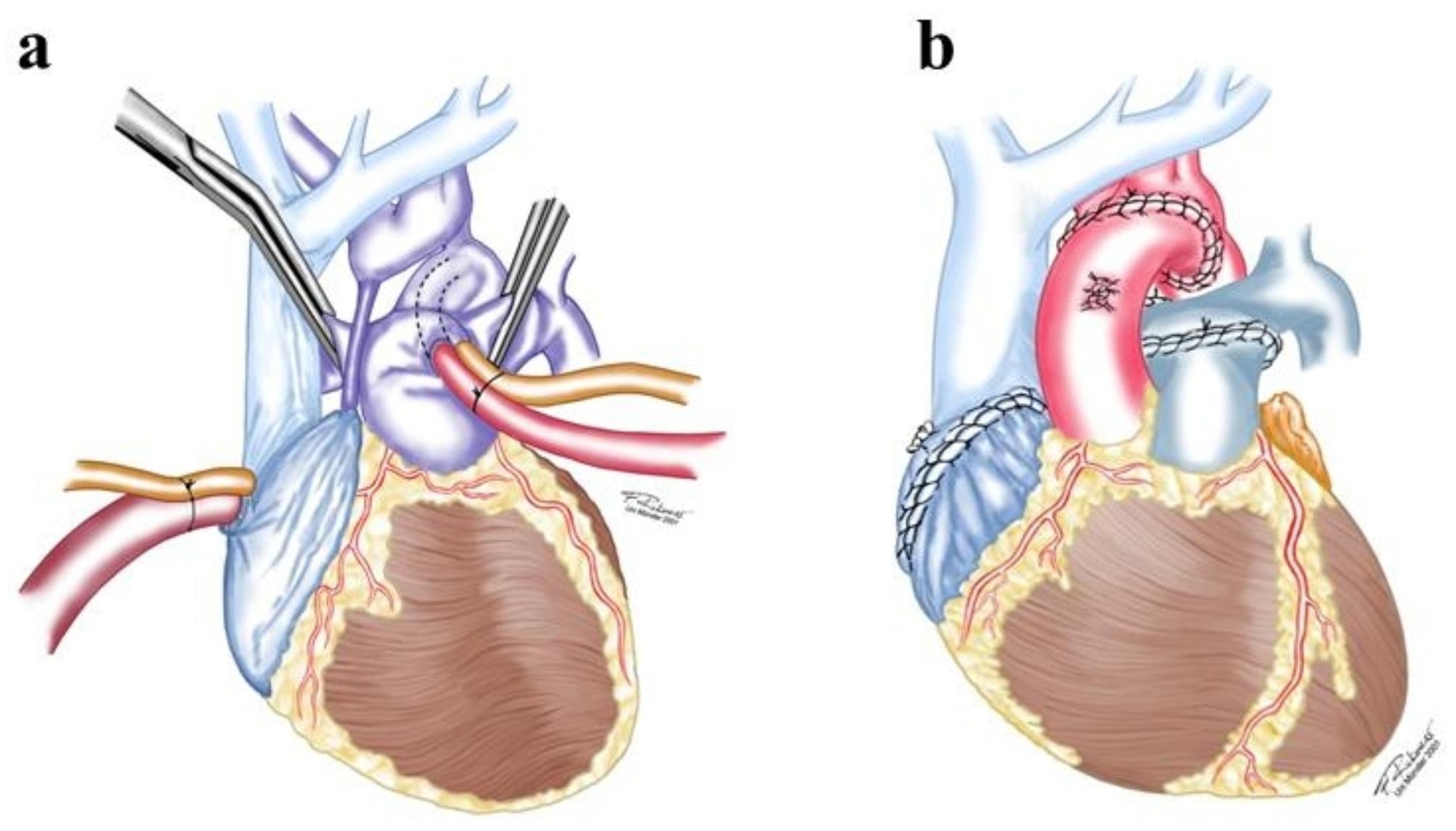
**(a)** Heart-lung machine connection for HLHS; **(b)** Anastomosis in heart transplantation for HLHS.

Extracorporeal life support (ECLS) use after cardiac surgery is often initiated to stabilize oxygenation. Concerning the survival of infant CHD patients on the waiting list, ECLS support remains inferior to VAD [14]. Figure 1.

Recent studies indicated that CHD patients with VAD support have longer waiting list times and are less likely to have a transplant [32, 33]. CHD patients with VAD support lose their urgent priority status unless complications or progressive heart failure occur.

In a CHD neonate supported with VAD and listed with a high urgency status for transplantation in our cohort, the long waiting time of 452 days was also a piece of definite evidence [25]. These findings suggest that VAD should be considered a treatment option for CHD patients before heart transplantation, and a dedicated heart transplant guideline is advocated for CHD patients.

## Management of cardiopulmonary bypass in transplant patients with CHD

Heart transplantation has been widely performed, and the operating procedures have been standardized, even in complex anatomical abnormalities and after multiple previous cardiac surgeries. However, re-sternotomy is a challenge when extensive adhesions and cardiomegaly increase hemorrhage risk. If a substernal great vessel and conduit adhesions exist, precautionary cardiopulmonary bypass (CPB) via the peripheral vessels may be considered prior to sternotomy to manage emergency situations [34]. However, the peripheral vessels have a very small lumen in pediatric patients and may be narrow or distorted due to previous surgery or catheterization, which can be troublesome. Vessels therefore often have to be patched. Usually, vessel patency needs to be evaluated by echocardiography and additional contrast CT scan.

## Physiological and technical challenges related to patient pathology

Standard surgical procedures for orthotopic heart transplantation include bicaval, biatrial, and total anastomoses techniques. These methods need adjustments in most patients requiring transplantation for CHD due to abnormal vessel anatomies and distortions. The common adjusted procedures have been described in patients diagnosed with hypoplastic left heart syndrome (HLHS), Fontan circulation, fetal Marfan syndrome, after Mustard procedure and situs inversus. For satisfactory implantation of the donor’s heart and reconstruction, it is advisable to harvest extended portions of the great vessels and thus facilitate complex anastomoses or reconstructions. Extended donor cardiectomy can avoid prosthetic material use, which may increase bleeding and infection risks. Using the total orthotopic technique, a mismatch in size can be tolerated.

The special features of different pathologies are discussed in detail below.

### Ischemic and dilated cardiomyopathy

On patients with ischemic or dilated cardiomyopathy, if there are no other malformations, heart transplantation can principally be performed using the classical or modified (biatrial) Shumway technique [35, 36]. However, direct anastomoses of the pulmonary veins and inferior and superior venae cavae (IVC, SVC, bicaval technique) are nowadays state-of-the-art as less rhythm disturbances occur and the geometry of the recipients’ hearts remains physiological. Even slightly bigger hearts are suitable for transplantation [37].

### Hypoplastic Left Heart Syndrome (HLHS) and Fontan Circulation

In the past decade, palliative therapy via a series of three surgeries has improved clinical outcomes in patients with HLHS [38–41]. Patients usually end up with a Fontan circulation and all its potential risks. Multifactorial causes are involved in the subsequent failure of Fontan circulation.

The common characteristic of HLHS is a hypoplastic development of left-sided cardiac structures. During transplantation, cardiopulmonary bypass (CPB) needs to be established via ductus arteriosus and right atrium (RA) (Fig. 1a). During deep hypothermia, the donor’s heart can be transplanted, and the aortic arch and PA reconstructed (Fig. 1b).

**Fig. 2:**
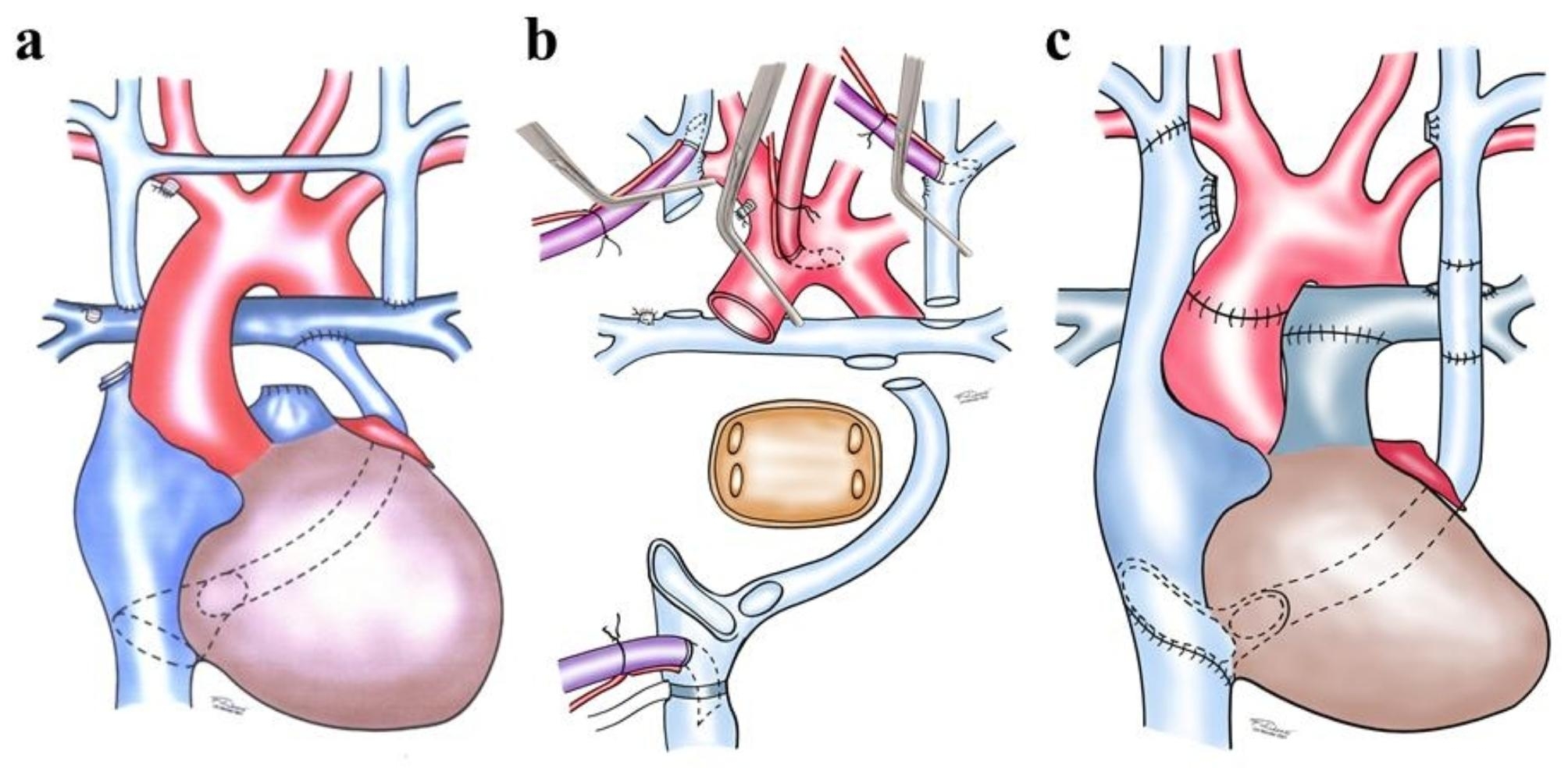
**(a)** Fontan circulation; **(b)** Situs after explantation; **(c)** Anastomoses for Fontan circulation

**Fig. 3:**
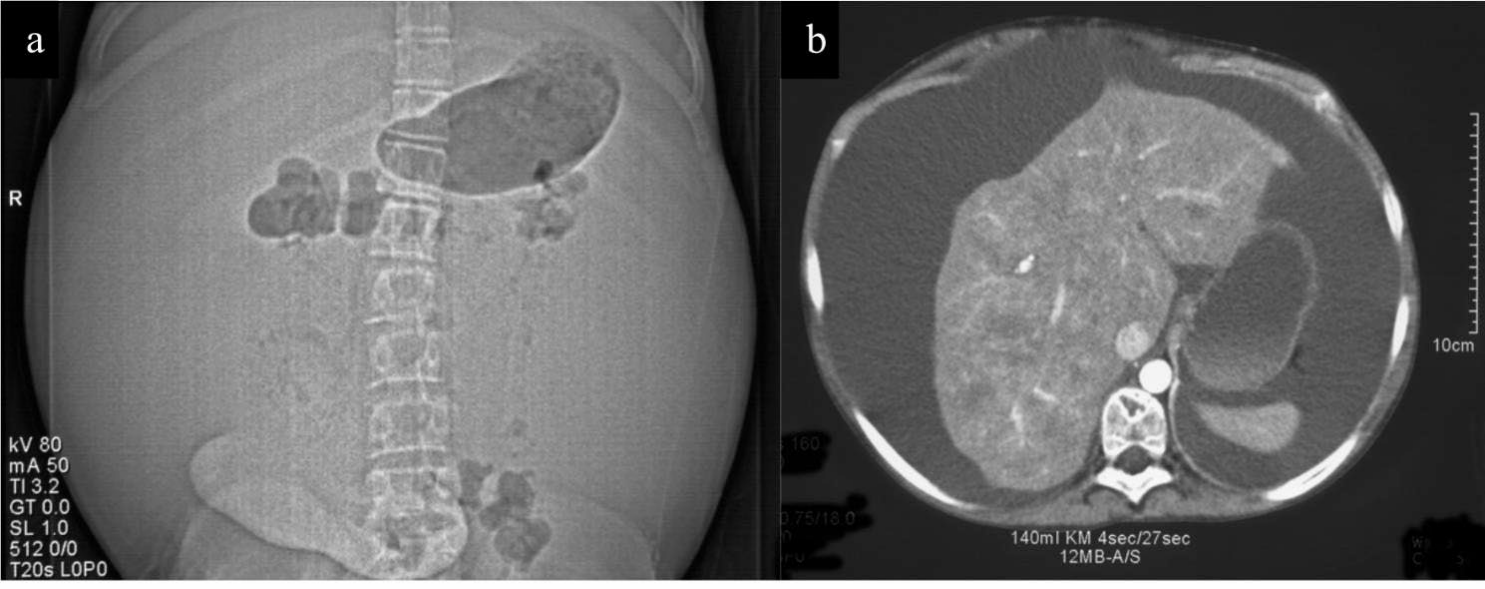
CT scan illustrating massive ascites due to enteropathy protein loss in a patient with Fontan circulation

We would like to underline the special challenges occurring with Fontan patients as we describe the procedure for a 20-year-old woman, originally born with a single ventricle (morphological right-ventricular type), persistent left SVC, and normally positioned arteries. PA banding and Blalock-Taussig shunt were applied at the age of five. A superior bidirectional cavo-pulmonary anastomosis was established two years later, and a patch was sewn in the RA to drain the IVC into the PA via the coronary sinus (Fig. 2a). At the age of 20, the young woman suffered from massive ascites (waist circumference: 83 cm) with enteropathy protein loss (Fig. 3).

As optimal venous drainage is essential for a successful surgery, CPB was established with one arterial cannula in the aorta and three venous cannulas in the IVC, right and left SVC (Fig. 2b).

The surgical procedure was performed stepwise: First, right and left atrium were resected, the posterior wall of the left atrium and coronary sinus were left in-situ. Second, the anastomosis between the right PA and right SVC was separated, and an anastomosis between the recipient’s and donor’s atrium was performed (bicaval technique). Then, after removing the previously inserted patch in the RA, the donor’s IVC was anastomosed with the recipient’s IVC and the coronary sinus. Third, the donor PA was anastomosed with the recipient’s PA bifurcation, and the donor aorta was connected with the ascending aorta before X-clamp removal. Fourth, the anastomosis between the left persistent vena cava and the coronary sinus was created on the beating heart. The recipient’s vascular openings after removing the cavopulmonary anastomoses and small obliterated vena anonyma were then sutured over (Fig. 2c).

If there is a lack of transplantable material, the donor’s heart must be removed with the brachiocephalic vein to ensure continuity between the left persisting SVC and the right SVC. If the donor’s brachiocephalic veins are too small, both donor’s and recipient’s brachiocephalic veins can be incised and anastomosed. Alternatively, a bovine jugular vein segment, such as Contegra^®^-graft can be used.

### Neonatal marfan syndrome

**Fig. 4:**
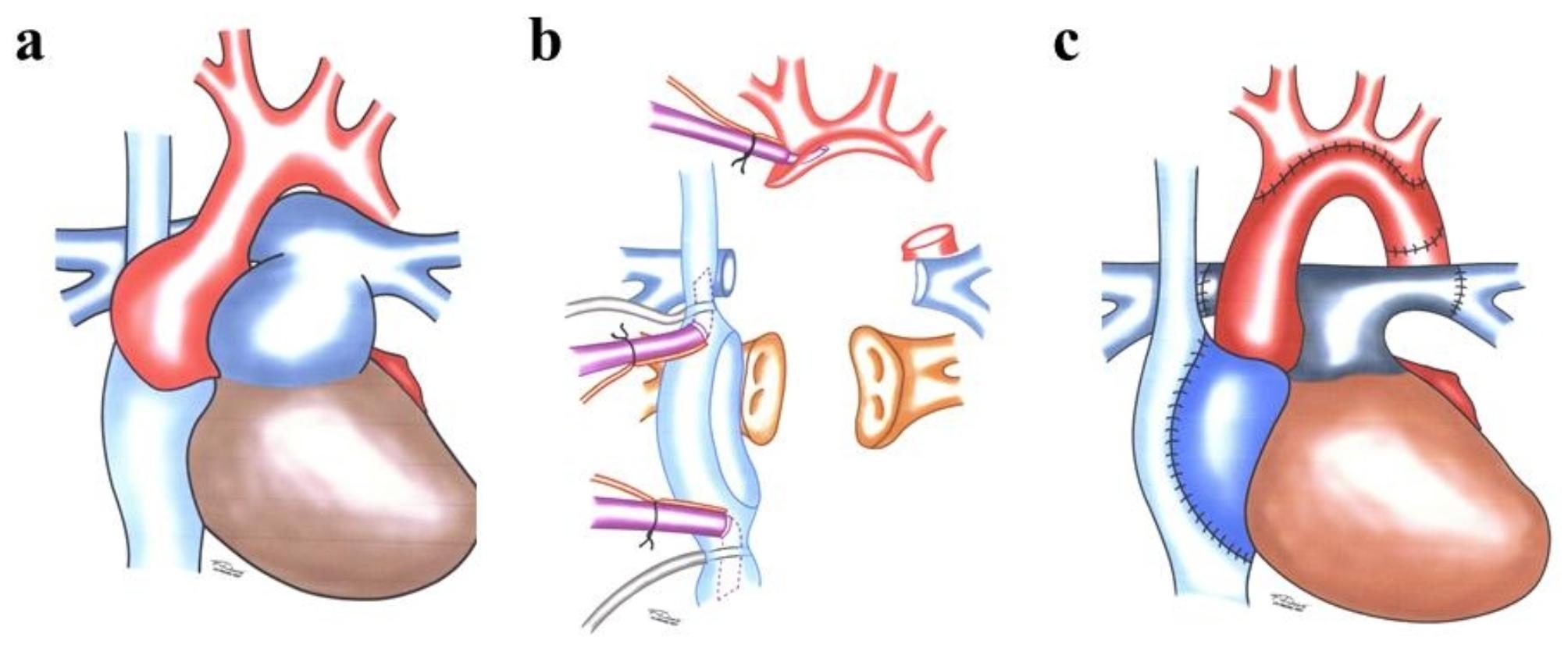
**(a)** Recipient situs in Marfan syndrome; **(b)** Situs after explantation; **(c)** Anastomoses for Marfan syndrome

Patients with neonatal Marfan syndrome have an extremely poor prognosis since heart valves insufficiencies lead to rapidly progressing heart failure. Heart-related vessels are often dilated (Fig. 4a).

Usually, patients undergo several operations [42–44] before they are listed for transplantation. The earlier the patients have a transplant the better their prognosis. It is crucial to transplant the adjoining large vessels. Therefore, the recipient’s heart and dilated parts of vessels should be resected in such a way (Fig. 4b) that the aortic arch and PA can be reconstructed with the donor’s vessels (Fig. 4c) [46].

### Status post mustard procedure

Until the early 1990’s, Mustard procedure as an atrial-level repair was the preferred surgical strategy for patients with a transposition of the great arteries until the arterial switch operation became the gold standard. Nowadays, there are still some patients after Mustard procedure who need transplantation as they suffer from severe systemic (right) ventricular failure and secondary pulmonary hypertension.

### Situs inversus

**Fig. 5:**
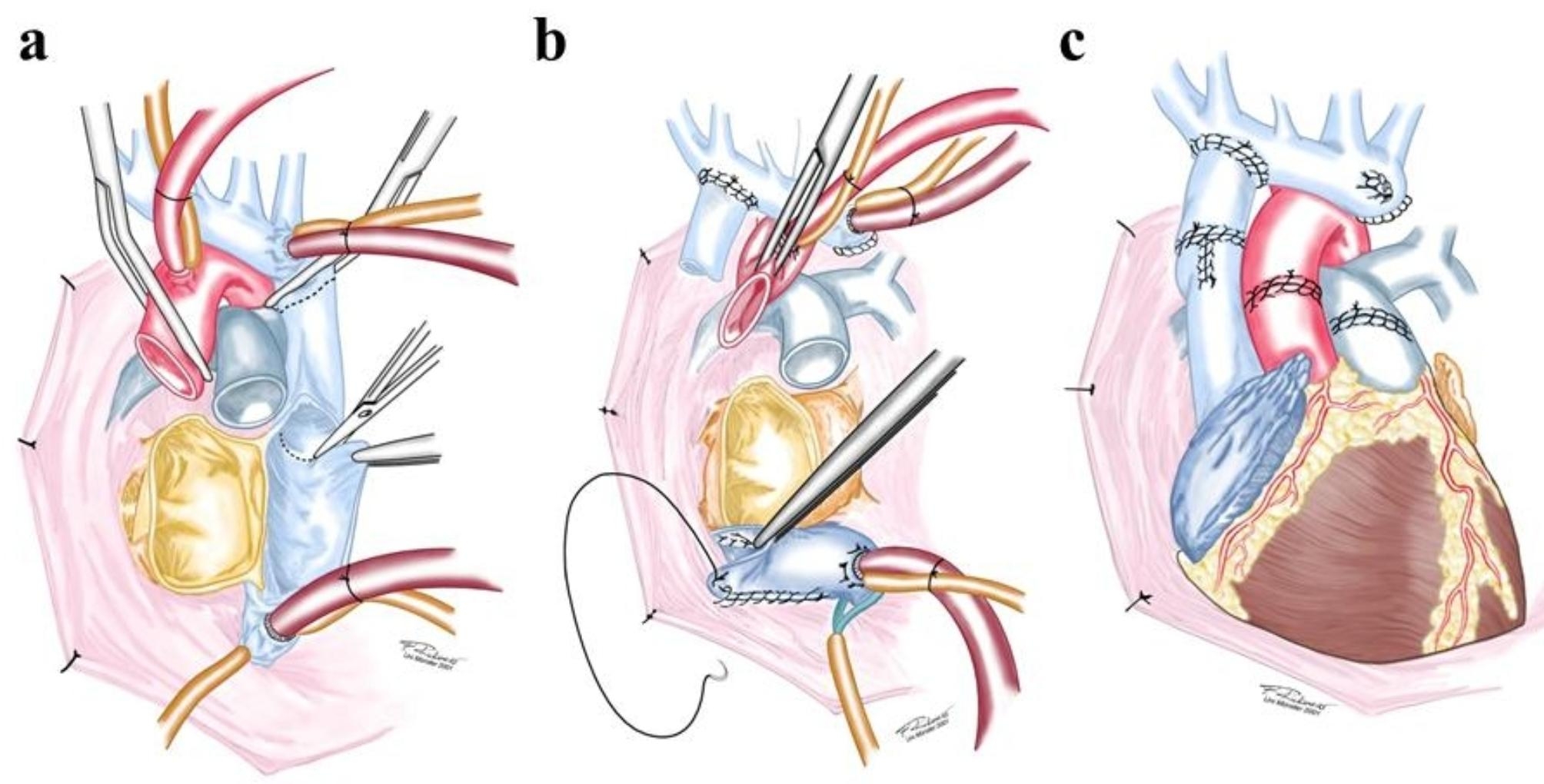
**(a)** Heart-lung machine connection for situs inversus and resection of left SVC; **(b)** Reconstruction of system venous drainage; **(c)** Anastomoses for situs inversus

Situs inversus describes a condition with a mirror-image reversal of the organs in the chest and abdominal cavity. It is often associated with CHD, mainly with transpositions of the great vessels. If patients with situs inversus suffer from end-stage heart failure, heart transplant can be the only curative treatment option, even after multiple previous operations [45, 46]. When removing the donor’s heart, adjacent vessels should be harvested as much as possible, especially SVC, brachiocephalic vein, aorta, and PA [45]. Heart transplantation is a great surgical challenge in situs inversus, aiming to rebuild a right-sided venous system with the left atrium and aorta in the middle. The remaining part of the recipient’s RA on the left needs to be separated and mobilized (Fig. 5a) to build a tunnel with the surrounding pericardium (Fig. 5b). Deep hypothermia and cardiac arrest can facilitate this anastomosis technique with a better operation field view after removing the lower venous cannula. The left SVC has to be anastomosed with the brachiocephalic vein (Fig. 5c). The pericardium must be incised to the left as far as the phrenic nerve to create enough space for the left ventricle [45, 46].

### Malignant cardiac tumors

Malignant cardiac tumors can also be an indication for heart transplantation. Two patients of this group have had a transplant in our center. They are not included in our analysis as their underlying diseases are not congenital heart defects in the classical sense [47].

## Outcomes and prognosis

Pediatric heart transplantation accounts for about 14% of total heart transplantations. The indication is changing with the development of palliative surgeries for CHD. Most frequent indication for listing remains progressive heart failure after prior surgeries (especially failing Fontan circulation) [48]. Published data reported a median post-transplanted survival of more than 15 years in pediatric patients who survived the first year after transplantation [49]. In detail, it showed median survivals of 22.3 years, 18.4 years, 14.4 years, and 13.1 years for patients < 1 year, 1 to 5 years, 6 to 10 years, and 11 to 17 years of age at transplantation, respectively [49]. Therefore, re-transplantation for these pediatric populations is more frequently expected and ultimately considered [50].

The perioperative mortality risk is exceptionally high. Based on the severe shortage of heart donors, giving these recipients scarce donor hearts calls the technique into question. Looking back at the early results, it is hard to tell how many patients died while they were listed for transplantation but it was a noteworthy number.

## The Muenster University experience

The first neonatal heart transplant at Muenster University Hospital took place in 1991. During the last 3 decades, 460 more heart transplantations were performed. 4.6% of these patients (n = 20, 9 males and 11 females) suffered from CHD with heterogeneous diagnoses. Mean age at the time of transplantation was 16.2 years, the youngest patient was 39 days, and the oldest 42 years old. Most of the CHD patients (60%) were children. Only three patients (15%) had no previous heart operation. 15 (75%) patients had a biventricular outflow tract, and five patients (25%) had a univentricular outflow tract. Seven patients (35%) underwent concurrent reconstruction procedures because of accompanied malformations, six (30%) received ventricular assist devices before transplantation.

Due to the complex cardiac malformations and frequent previous surgeries, four patients died within 30 days after heart transplantation. Especially, the shift from Fontan circulation to a biventricular circulation was a challenge and thus associated with a high early mortality rate. 2 out of 3 Fontan patients died due to lung and/or multiorgan failure. 16 patients (80%) were finally discharged post transplant. 45% of the transplanted patients needed re-do procedures but no patient needed to be re-transplanted within the last 20 years. 3 Patients developed hemorrhagic shock due to hemothorax early after transplant and re-exploration to assess for bleeding was required. 1 patient suffered from diaphragmatic paresis that needed surgical treatment and 2 others underwent secondary chest closure.

Even the first small children who were transplanted in 1988 [6] were still alive with their first donor hearts when the follow-up ended in April 2022.

Our results showed an acceptable early mortality compared to other working groups, while compared to conventional transplantation, early mortality was relatively high. However, given this historical patient population and the surgical and intensive care capabilities of that time, the outcome of the earlier days was remarkable.

## Discussion

Heart transplantation is a bail-out strategy for patients who cannot profit from any other medical/surgical therapy. Despite many advances in surgical procedures and perioperative management, early mortality after transplantation remains higher in CHD patients than in patients with non-CHD heart disease. Simultaneously, a comparable survival and even superior benefit can be observed in CHD patients compared with non-CHD patients [51]. Though the number of transplants in adult patients with CHD remains low, there is a steadily increasing trend in recent years [52]. Due to the shortage of donor hearts for children and improved survival with corrective and palliative interventions, the number of heart transplants for pediatric CHD has decreased [22, 53].

Cardiac transplantations during childhood have immunological benefits, especially during the first 30 days of life. Because of the immature immune system in the pediatric population, the ABO-incompatible transplantation remarkably reduces the waiting list time and expands the potential donor pool for pediatric patients. Besides, an international multicenter study with 58 ABO-incompatible heart transplantations revealed a satisfied long-term prognosis with freedom from death or re-transplantation of 100%, 96%, 69% at 1, 5, 10 years, respectively [54]. Re-transplantations represent approximately 5% of all pediatric heart transplantations. However, long-term survival after second transplantation is significantly lower in comparison to primary transplantation [50]. MCS use in pediatrics is encouraged for its benefits.

Many factors contribute to the early mortality after heart transplantation in CHD patients - including failing Fontan pathophysiology, prolonged ischemia time due to the complex anatomy, increased pulmonary pressure, and older recipient age [55]. Nevertheless, many studies found a similarly good or even better long-term survival than “conventional” transplantation when patients survived the early stage [51, 56]. Good long-term results can only be achieved through intensive interdisciplinary cooperation between cardiologists, grown-up congenital heart specialists, and surgeons.

In the current study, we gave a comprehensive description of heart transplantation surgery in different CHD scenarios. Besides, consistent with previous studies [22, 51], our single-center data and follow-up results demonstrated that the early mortality is significant, but the long-term prognosis was satisfactory in CHD patients. We highlight the particularity of these patients and the anatomical and physiological impact on heart transplant surgeries.

In summary, considering the severity of illness in CHD patients, higher early mortality but comparable long-term survival show that heart transplantation is an effective treatment option. Though heart transplantation procedures have been standardized, adjustments of complex anastomoses or reconstruction are very common due to CHD patients’ special characteristics. Cardiovascular imaging, such as catheterization, computer tomography scan, or magnetic resonance imaging, should be performed in patients with complex congenital anatomy. 3D printed anatomical heart models should be encouraged because of their direct intuitive exhibition.

As for the donor heart shortage, VAD should be considered a treatment option, and a dedicated heart transplant guideline is advocated for CHD patients supported with VAD.

## Data Availability

The data that support the findings of this study are available from the corresponding author, AH, upon reasonable request.
